# Commercial phenoxyacetic herbicides control heavy metal uptake by wheat in a divergent way than pure active substances alone

**DOI:** 10.1186/s12302-017-0124-y

**Published:** 2017-09-28

**Authors:** Elżbieta Skiba, Wojciech M. Wolf

**Affiliations:** Institute of General and Ecological Chemistry, Lodz University of Technology, Żeromskiego 116, 90-924 Łódź, Poland

**Keywords:** Commercial herbicide formulations, 2,4-D, MCPA, Aminopielik, Chwastox, *Triticum aestivum* L., Heavy metals bioaccumulation and translocation

## Abstract

**Background:**

Impact of two widely used commercial herbicides, i.e. Aminopielik D 450 SL and Chwastox 300 SL, on the uptake and translocation of selected heavy metals in wheat plants *Triticum aestivum* L. cultivated in the laboratory pot experiments was investigated. Mineral-humus, loamy sand soil representative for the central part of Poland was applied. Bioavailable, exchangeable and total forms of Cd, Co, Cu, Zn, Pb, and Mn were determined. Transfer coefficients, translocation, and bioaccumulation factors illustrating metal migration in the plant were investigated.

**Results:**

Administration of commercial herbicides significantly altered heavy metals uptake by wheat in a way distinctively different than that observed for the parent chemically pure synthetic auxins, i.e. 2,4-D and MCPA. In particular, Aminopielik D 450 SL and Chwastox 300 SL prompted heavy metals accumulation in roots as indicated by their high transfer coefficients. Further transport to above ground part of the plant was limited and element dependent.

**Conclusions:**

This work clearly shows that commercial herbicide formulations may act in a distinctively different way than pure active ingredients alone.

**Electronic supplementary material:**

The online version of this article (doi:10.1186/s12302-017-0124-y) contains supplementary material, which is available to authorized users.

## Background

Commercial herbicides are rarely pure active compounds. Nowadays, commonly used formulations contain supplementary ingredients which work as carriers, surfactants, stabilizers or dyes [1]. Those which enhance biological activity of herbicide are called adjuvants [2]. They are classified as activators and spray or utility modifiers and are added to herbicide either at the factory or field level. Today, more than 3000 adjuvants of diverse constituencies are offered on a market [3]. Additionally, formulations contain safeners which increase the herbicide selectivity between crop and weed species [4]. They show a wide range of affinities and selectivities towards heavy metals in soil. In particular, amines which are frequently present in commercial products are prone to interact with metals [5] and influence their uptake and absorption by the plant roots. Regrettably, investigations on heavy metal accumulation by agricultural plants under the presence of herbicides are focused on pure active substances [6]. Commercial formulations are examined scarcely [7]. This is difficult because according to current EU and US regulations [8, 9] chemical composition of additives is not to be mentioned on the product label and is usually bottled up by the manufacturers. They are obliged to specify the active substances of particular herbicide formulation only. This is a very uncomfortable situation because all ingredients are being introduced to the environment and their join impact cannot be properly assessed. Moreover, toxicity evaluations based on a single component can often lead to misleading conclusions [10]. The latter was proved by Mesnage et al. [8] who found that in certain circumstances organic adjuvants present in glyphosate-based herbicides were 10,000 times more toxic than active substance. We believe that this situation ought to be changed at the national and the EU legal level and manufacturers should publish the complete composition of all formulations in the market. Phenoxyacetic herbicides are synthetic auxins widely used in agriculture. They control broadleaf weeds by affecting growth of the plant’s vascular tissue and are being often applied to protect grass and grain crops [11]. The most common representatives are 2,4-dichlorophenoxyacetic acid (2,4-D) and 2-methyl-4-chlorophenoxyacetic acid (MCPA) [12]. They are active ingredients of two agrochemicals widely used in Europe, i.e. Aminopielik D 450 SL and Chwastox 300 SL.

In current work, impact of these two commercial formulations on the accumulation and distribution of heavy metals in *Triticum aestivum* L. plants was analysed and further compared to that induced by pure sodium salts of 2,4-D and MCPA as recently published by Skiba et al. [13]. To make this evaluation feasible, the soil and cultivation conditions were identical during this and former studies. To our knowledge, this is the first experimental work on the heavy metals uptake by wheat originated by commercial phenoxyacetic herbicide formulations widely used in agriculture.

## Methods

### Soil

The mineral-humus, loamy sand soil [14] was used for wheat cultivation. Samples were collected from the rural environment in the central Poland Skierniewice region (51°97′N, 20°07′E). Procedure as described in the standard PN ISO 10381–4 was applied [15]. The same soil was used by us in the study of the heavy metals uptake induced by chemically pure 2,4-D and MCPA [13]. Soil was air-dried and sifted through a sieve with 2 mm mesh diameter and stored in plastic container. Soil pH was measured by the potentiometric method in 1 mol L^−1^ potassium chloride solution [16]. The gravimetric method for the determination of soil organic matter by the mass loss at 550 °C was applied [17]. The grain size analysis was performed according to the Polish Standard PN-R-04032 [18]. Exchangeable and bioavailable forms of metals were determined in 1 mol L^−1^ solutions of MgCl_2_ and HCl, respectively [19]. The total content of metals was evaluated by the soil decomposition method employing mixture of H_2_SO_4_ and HF [20].

### Herbicides

Two commercial herbicide formulations were used in this study, namely Aminopielik 450 SL (PCC Rokita S.A.) and Chwastox 300 SL (Ciech Sarzyna S.A.). Subsequently, they will be abbreviated as Aminopielik and Chwastox, respectively. The former contains in 1 L 417.5 g 2,4-D and 32.5 g dicamba (3,6-dichloro-2-methoxybenzoic acid) in the form of dimethylammonium salts. The latter is the sodium–potassium salt of MCPA supplied at the concentration 300 g L^−1^. Spray loads followed the field application rates as recommended by manufacturers, i.e. 3 L ha^−1^. They correspond to 1.3 and 1.0 kg ha^−1^ of chemically pure 2,4-D and MCPA, respectively.

### Wheat cultures

High-quality Rywalka winter wheat (*Triticum aestivum* L.) seeds from PPH „Rolpuch” Co. LTD Kutno were used in the study. All plants were grown in pot experiments under the laboratory conditions following the procedure developed by Kobyłecka and Skiba [21]. Dry weight 400 g portions of soil were placed in rectangular (17 × 11 cm) plastic pots of the height 6 cm. The soil was further watered with 100 mL of deionized water. Wheat seeds (20 g) were sowed and covered with additional 100 g of soil. Pots were watered to keep the soil moisture in the range 5–10% and kept at temperature 20 °C. Cultivation experiments were performed in two arrangements, each was related to one type of herbicide formulation. The third arrangement was cultivated as a reference without the addition of herbicides. A single batch consisted of cultures with five pots giving fifteen samples altogether. Sprays were applied in a week after sowing when the plants were in a stage 11 of either the Zadoks et al. [22] or BBCH [23] growth scales. Cultures were terminated after 3 weeks of growth (stage 13). The above ground parts of plants were cut while the roots were separated from soil by washing and rinsing with distilled water. The harvest was dried at 55 °C, homogenized and further ground.

### Determination of metals in wheat shoots and roots

The weighted plant subsamples (0.7 g—shoots and 0.5 g—roots) were digested in the mixture of concentrated HNO_3_ (65%) and H_2_O_2_ (30%) (3:1, v/v) using the Anton Paar Multiwave 3000 closed system instrument. The content of Cu, Zn and Mn was determined by the FAAS (GBC 932 plus) while Pb, Cd and Co were analysed by the GFAAS (GBC, SensAA). The reliability of analytical procedures was checked using certified references materials: INCT-MPH-2 [24] and IAEA-V-10 [25].

### Statistical analysis

A one-way analysis of variance ANOVA as implemented in OriginPro 2016 was used to test the impact of Aminopielik and Chwastox at 0.95 probability level. Numerical data are available from the authors on request.

### Bioaccumulation, translocation and transfer factors

Metal migration was evaluated by the transfer coefficient (TC) and bioaccumulation factor (BAF). They are ratios of particular element concentration in either root or shoot, related to its content in the soil [26]. Metal distribution inside plant body was assessed by the translocation factor (TF) which is the ratio of element concentration in above ground part of the plant to that in roots [27].

## Results and discussion

**Fig. 1:**
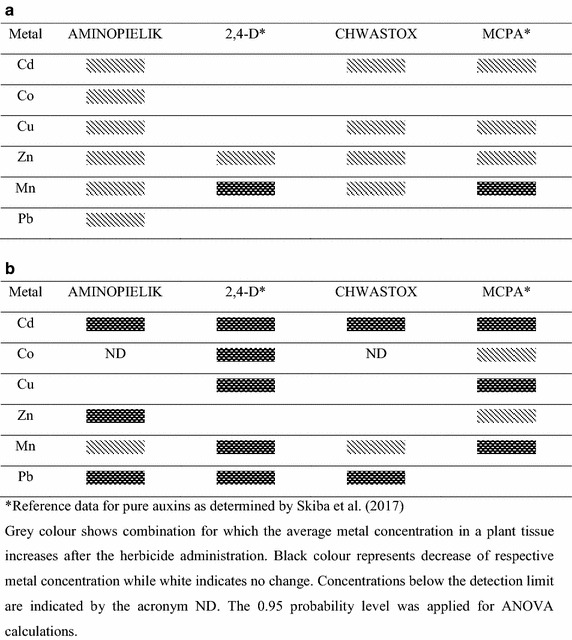
The influence of Aminopielik and Chwastox on heavy metals accumulation in wheat roots (**a**) and shoots (**b**). Asterisk reference data for pure auxins as determined by Skiba et al. [13]. Grey colour shows combination for which the average metal concentration in a plant tissue increases after the herbicide administration. Black colour represents decrease of respective metal concentration while white indicates no change. Concentrations below the detection limit are indicated by the acronym ND. The 0.95 probability level was applied for ANOVA calculations

Representative farmland soil from the central Poland was used in this study. Concentrations of metal forms as summarized in Additional file 1: Table S1 do not exceed the maximum allowable concentrations (MAC) and trigger action values (TAV) tabulated by Kabata-Pendias and Mukherje [28]. Bioavailable forms were identified for all metals with the largest contribution observed for lead and manganese. Exchangeable components of all species except cobalt were visible. The impact of Aminopielik and Chwastox on heavy metals accumulation was evaluated by the one-way ANOVA. Relevant data for chemically pure auxins 2,4-D and MCPA as determined by Skiba et al. [13] are given for comparison (Fig. 1). Metal contents in roots and shoots of wheat cultivated without and with herbicides treatment are given in the Additional file 2: Table S2. Aminopielik elevated concentrations of all metals in roots while 2,4-D was successful for zinc only. Cadmium, cobalt, copper and lead were left unchanged while the manganese content decreased upon the pure auxin administration. Chwastox increased cadmium, copper, zinc and manganese levels in roots, while cobalt and lead were left unchanged. Content of manganese decreased upon the MCPA treatment. More complicated picture was observed for shoots. Aminopielik decreased cadmium, zinc and lead concentrations. Copper level was unchanged while that of manganese was increased. The 2,4-D administration resulted in decreasing contents of all metals except zinc. Chwastox treatment gave effects distinctively different than those of MCPA for all metals except cadmium. Cobalt and zinc contents increased while copper and manganese levels decreased; lead was unchanged. On the contrary to pure 2,4-D and MCPA, both commercial herbicides prompted higher heavy metals accumulation in roots. This metal preconcentration was the most apparent for zinc as emphasized by its TCs which were higher than unity (1.75, 2.27 for Aminopielik and Chwastox, respectively). Series of metals ordered according to decreasing TCs, TFs and BAFs calculated for plants treated with Aminopielik and Chwastox are summarized in Table 1; numerical data are in Additional file 3: Figure S1. Transfer coefficients determined for plants cultivated in the reference, untreated soil are in the order Zn > Cd > Cu > Pb > Co > Mn. Administration of Aminopielik, 2,4-D or Chwastox left that arrangement untouched. Treatment with the pure MCPA interchanged that order for lead and cobalt only. Nevertheless, commercial herbicides increased TCs for all metals investigated as compared to chemically pure auxins. Migration of metals in the plant body may be conveniently examined with the translocation factors. However, the emerging picture is more complex than that observed in roots. TFs for pure 2,4-D and MCPA are in the order Mn > Cu > Zn > Cd > Co > Pb and Mn > Pb > Cu > Zn > Cd > Co, respectively. Aminopielik administration alters that arrangement for Pb, Cd and Co while Chwastox interchanges positions of Cu and Pb only. Influence of Aminopielik on heavy metals uptake by roots is more pronounced than that of Chwastox. However, impact of both herbicides is much less clear in the above ground parts of the wheat plant. Both formulations hampered zinc transport to above ground parts of wheat and stabilized its accumulation in roots. The opposite effect was observed for manganese. Either Aminopielik or Chwastox mobilized this element within the plant body and significantly increased its concentration in shoots. On the contrary, either 2,4-D or MCPA administration led to accumulation and stabilization of manganese in roots alone. Aminopielik increased the lead accumulation in roots and reduced its further migration to the upper part of the plant in a more pronounced way than that of Chwastox. Variation of copper content in shoots was quite limited over all samples and followed the well-recognized low mobility of this element [29]. Cobalt and cadmium were scarcely present in soil. However, their uptake was facilitated by either Aminopielik or Chwastox and restricted to roots only.

**Table 1:** Series of metals ordered according to decreasing transfer coefficients (TC), translocation (TF) and bioaccumulation (BAF) factors

Treatment	TC	TF	BAF
Aminopielik	Zn > Cd > Cu > Pb > Co > Mn	Mn > Cu > Zn > Pb > Cd > Co^a^	Zn > Cu > Mn > Cd > Pb > Co^a^
2,4-D	Zn > Cd > Cu > Pb > Co > Mn	Mn > Cu > Zn > Cd > Co > Pb	Zn > Cd > Cu > Mn > Pb > Co
Chwastox	Zn > Cd > Cu > Pb > Co > Mn	Mn > Cu > Pb > Zn > Cd > Co^a^	Zn > Mn > Cu > Cd > Pb > Co^a^
MCPA	Zn > Cd > Cu > Co > Pb > Mn	Mn > Pb > Cu > Zn > Cd > Co	Zn > Cd > Cu > Pb > Co > Mn
Reference	Zn > Cd > Cu > Pb > Co > Mn	Mn > Pb > Cu > Cd > Zn > Co	Cd > Zn > Cu > Pb > Mn > Co

^a^Co content in shoots was below the detection limit (1 ppb); therefore, it was assumed that respective TFs and BAFs are zero

Results for untreated reference sample, 2,4-D and MCPA [13] are included for comparison

## Conclusions

This work is based on a more comprehensive approach to herbicide activity than that described by Skiba et al. [13]. On the contrary to that publication on the metal uptake upon the pure herbicide treatment, in this paper the special emphasis is put on the activity of commercial formulations practically used in agriculture. This issue is of particular relevance in view of increasing number of publications focused on toxicity originated from agrochemical ingredients and additives [30]. All living organisms may be affected with bees and other pollinators being the most prone [31–33].

Administration of two investigated commercial herbicide formulations significantly altered the heavy metals uptake by wheat. Their influence was distinctively different than that observed for the parent chemically pure synthetic auxins, i.e. 2,4-D and MCPA. In particular, they prompted higher metal uptake and their further accumulation in roots. However, the subsequent metals migration to the upper part of the plant was controlled by Aminopielik and Chwastox in a less predictable way. The highest influence was observed for manganese and zinc while cadmium content in shoots decreased regardless to the type of applied herbicide.

Our results are in line with recent publications reporting that adjuvants activity extends far beyond the simple mechanics of herbicide application and adhesion. They may also affect biochemical processes responsible for metal uptake and translocation [34]. We therefore postulate that the usual activity and toxicity tests would involve herbicide formulations used in contemporary agriculture and not to be limited to their active ingredients alone. Moreover, the EU legislation and national regulations should bind the herbicide manufacturers and suppliers to publish the complete composition of all formulations they introduce in the market.

## Additional files

**Additional file 1: Table S1.** General properties of soil (mean ± SD, n = 3) (a), Metal contents (mg kg^−1^) in soil (mean value ± SE, n = 3).

**Additional file 2: Table S2.** Metal contents with SE (mg kg^−1^) in roots and shoots of wheat cultivated without and with herbicides treatment.

**Additional file 3: Figure S1.** Transfer coefficients (TC) (a), translocation factors (TF) (b) and bioaccumulation factors (BAF) (c) of heavy metals in wheat untreated and under Aminopielik or Chwastox administration.

## Abbreviations

2,4-D: 2,4-dichlorophenoxyacetic acid
MCPA: 2-methyl-4-chlorophenoxyacetic acid
BBCH: Biologische Bundesanstalt, Bundessortenamt und CHemische Industrie Cereal growth staging scales
FAAS: flame atomic absorption spectrometry
GFAAS: graphite furnace atomic absorption spectrometry
ANOVA: a one-way analysis of variance
