# Supplementary material for: Commercial phenoxyacetic herbicides control heavy metal uptake by wheat in a divergent way than pure active substances alone
Source: Environ Sci Eur. 2017 Sep 28;29(1):26. doi: 10.1186/s12302-017-0124-y (PMC5617864; doi:10.1186/s12302-017-0124-y)
Supplement: Supplementary file 1 — Additional file 1: Table S1. General properties of soil (mean ± SD, n = 3) (a), Metal contents (mg kg−1) in soil (mean value ± SE, n = 3). [file 12302_2017_124_MOESM1_ESM.docx]

Additional file 1: Table S1. General properties of soil (mean ± SD, n = 3) (a), Metal contents (mg·kg^-1^) in soil (mean value ± SE, n = 3)

a)

| Reaction | | Total organic carbon  % | Granulometric composition  % | | |
| --- | --- | --- | --- | --- | --- |
| pH – H_2_O | pH – KCl |  | 1. – 0.1   mm | - 1. – 0.02   mm | < 0.02  mm |
| 6.31 ± 0.02 | 5.02 ± 0.01 | 4.03 ± 0.11 | 90.56 ± 0.81 | 9.18 ± 0.09 | 0.25 ± 0.04 |

b)

|  |  | Metal | | | | | |
| --- | --- | --- | --- | --- | --- | --- | --- |
|  | Forms | Cd | Co | Cu | Zn | Mn | Pb |
| Content  mg·kg^-1^ | Total | 1.15 ± 0.06 | 1.75 ± 0.16 | 94.0 ± 2.6 | 167 ± 1 | 351 ± 9 | 36.9 ± 0.7 |
|  | Bioavailable | 0.47 ± 0.03 | 0.74 ± 0.02 | 28.9 ± 1.8 | 49.9 ± 2.8 | 208 ± 4 | 25.8 ± 1.4 |
|  | Exchangeable | 0.24 ± 0.01 | ND | 0.33 ± 0.01 | 15.5 ± 0.3 | 6.17 ± 0.44 | 1.07 ± 0.08 |

ND – metal content below the detection limit (1 ppb)
