# Supplementary material for: Commercial phenoxyacetic herbicides control heavy metal uptake by wheat in a divergent way than pure active substances alone
Source: Environ Sci Eur. 2017 Sep 28;29(1):26. doi: 10.1186/s12302-017-0124-y (PMC5617864; doi:10.1186/s12302-017-0124-y)
Supplement: Supplementary file 2 — Additional file 2: Table S2. Metal contents with SE (mg kg−1) in roots and shoots of wheat cultivated without and with herbicides treatment. [file 12302_2017_124_MOESM2_ESM.docx]

Additional file 2: Table S2. Metal contents with SE (mg·kg^-1^) in roots and shoots of wheat cultivated without and with herbicides treatment

| Metal | Herbicide | Roots  mg·kg^-1^ | Shoots  mg·kg^-1^ |
| --- | --- | --- | --- |
| Cd | —  Aminopielik  Chwastox | 0.67 ± 0.03  1.50 ± 0.07  1.16 ± 0.04 | 0.38 ± 0.03  0.13 ± 0.02  0.15 ± 0.02 |
| Co | —  Aminopielik  Chwastox | 0.22 ± 0.01  0.34 ± 0.02  0.26 ± 0.01 | 0.03 ± 0.01  ND  ND |
| Cu | —  Aminopielik  Chwastox | 19.5 ± 0.1  37.7 ± 2.0  26.8 ± 0.9 | 12.7 ± 0.1  14.2 ± 0.6  13.6 ± 1.4 |
| Zn | —  Aminopielik  Chwastox | 110 ± 1  293 ± 7  379 ± 5 | 53.0 ± 1.2  50.0 ± 0.1  53.3 ± 0.1 |
| Mn | —  Aminopielik  Chwastox | 22.9 ± 2.3  50.3 ± 3.0  47.4 ± 1.8 | 17.7 ± 0.1  50.6 ± 1.6  50.8 ± 1.9 |
| Pb | —  Aminopielik  Chwastox | 4.73 ± 1.11  8.13 ± 0.42  6.19 ± 0.03 | 3.45 ± 0.61  0.91 ± 0.06  0.91 ± 0.04 |

ND – metal content below the detection limit (1 ppb)

Active ingredients of Aminopielik and Chwastox are 2,4-D and MCPA, respectively.
