# Supplementary material for: Commercial phenoxyacetic herbicides control heavy metal uptake by wheat in a divergent way than pure active substances alone
Source: Environ Sci Eur. 2017 Sep 28;29(1):26. doi: 10.1186/s12302-017-0124-y (PMC5617864; doi:10.1186/s12302-017-0124-y)
Supplement: Supplementary file 3 — Additional file 3: Figure S1. Transfer coefficients (TC) (a), translocation factors (TF) (b) and bioaccumulation factors (BAF) (c) of heavy metals in wheat untreated and under Aminopielik or Chwastox administration. [file 12302_2017_124_MOESM3_ESM.docx]

Additional file 3: Figure S1.Transfer coefficients (TC) (a), translocation factors (TF) (b) and bioaccumulation factors (BAF) (c) of heavy metals in wheat untreated and under Aminopielik or Chwastox administration

a)

**TC**

**TC**

b)

c)
